# Transfer of Promotion Effects on Elderly Health with Age: From Physical Environment to Interpersonal Environment and Social Participation

**DOI:** 10.3390/ijerph16152794

**Published:** 2019-08-05

**Authors:** Zhenhua Zheng, Hong Chen, Liu Yang

**Affiliations:** 1 College of Architecture and Urban Planning, Tongji University, 1239 Siping Road, Shanghai 200092, China; 2 College of Architecture and Environment, Sichuan University, No.24 First South Section First Ring Road, Chengdu 610065, China; 3 Institute of Local Governance, Yangtze Normal University, 16 Juxian Avenue, Chongqing 408100, China; 4 Center for Population and Development Policy Studies, Fudan University, 220 Handan Road, Shanghai 200433, China

**Keywords:** elderly health, physical environment, interpersonal environment, social participation, age differences

## Abstract

An important goal of building “age-friendly communities” is to help the elderly to access more opportunities for social participation and better health. However, little is known about the complex relationships between neighborhood environment, social participation, and elderly health. This study examined the mediating role of social participation in the area of neighborhood environment affecting elderly health and explored the discrepancy among different age groups in 43 neighborhoods of Shanghai. Both neighborhood environment and social participation had significant positive effects on elderly health in all the samples. Meanwhile, social participation served as a mediator of the relationship between interpersonal environment and elderly health. Furthermore, remarkably, health promotion effects transferred from the physical environment to interpersonal environment and social participation with age; the influence of physical environment on elderly health decreased with the increase of age, while the influence of interpersonal environment and social participation on the health of the elderly increased with the increase of age. This study found that physical environment, interpersonal environment, and social participation had different effects on elderly health of different ages. Different policies should be applied toward improving the interpersonal environment, optimizing of physical environment, and guiding the community activities.

## 1. Introduction

With the increasing degree of global aging, the international society’s concept of responses has also experienced a change from “successful aging” to “healthy aging” to “positive aging.” The role of the elderly should also change from “passive dependent” to “active participants in social activities” where social participation becomes an important part of positive aging [1,2,3]. In 2007, the World Health Organization (WHO) formally introduced the concept of “age-friendly communities,” which aims to encourage governments to create communities that can lead to greater participation of the elderly in more social activities and contribute to their health [4,5,6]. The concept emphasizes that community is the main activity place and living space for the elderly [7,8,9], and its environment plays an important role in the social participation and health of the elderly [10,11,12,13].

Some studies have confirmed the significant effects of the neighborhood environment on elderly health [14,15,16], and some others have explored the important impacts of social participation on elderly health [17,18,19]. Furthermore, the relationship between neighborhood environment, social participation, and elderly health has become one of the important directions of environmental gerontology [20,21].

The theoretical framework of this study is based on the “social ecology” theory, which emphasizes the interactions between environment, human behavior, and health [21,22,23]. In general, the neighborhood environment does not affect health independently, but through its influence on behavior. With the development of positive aging, more and more scholars are paying attention to the application of social ecology in neighborhood environment research. However, most studies have focused on the outdoor activity as a mediator of the relationship between the neighborhood environment and health of the elderly [24,25,26]. Social participation is a behavior that is very helpful in achieving positive aging. The role of social participation in the area of neighborhood environment that affects the health of the elderly has also been recognized by scholars. The study by Richard et al. [27] showed that the degree of social participation is closely related to the distance to the convenient resources of the living environment, and the built environment plays a potential role in social participation, which is part of health-relevant behaviors. Menec et al. [28] pointed out that from an ecological perspective, the roles such as community physical environment, participation opportunities, etc., that affect the health of the elderly are interrelated and holistic. Only a few studies have considered the issue, based on data from older people in Shanghai, which explored the relationships between the neighborhood environment, lifestyle, and health of older adults [29].

Meanwhile, because of the differences in the physical function, behavioral habit, and psychological state of older adults in different age stages, the effects of neighborhood environment and social participation on their health are also different. Many scholars agreed that social participation can promote the health of the elderly effectively [18,19,21,22]. However, most of the studies treated the older adults indiscriminately and rarely see the difference between the groups in different age stages.

In recent years, China has been facing the world’s largest and fastest-growing population aging [30], but little research has been done on the relationship between neighborhood environment and health of the elderly based on Chinese samples. Shanghai is not only China’s economic and financial center, but also one of the most rapidly aging cities, with 14.3 percent of the population aged 65 and over, and that proportion is expected to rise to nearly 20 percent by 2030 [31]. Therefore, the research based on the data collected in Shanghai can represent the current situation of China to some extent.

Based on the data from a large-scale sample survey of the elderly in Shanghai conducted by Fudan University in China, in this study, we constructed a concept model of “neighborhood environment–social participation–elderly health” by using the structural equation model (SEM). By exploring the relationships between neighborhood environment, social participation, and elderly health, and comparing the differences in this relationship among older adults at different age stages, our study is expected: (1) to enrich the research conclusions on the factors affecting elderly health, (2) to supplement the relevant conclusions from Chinese data, (3) to provide a reference for the detailed design of the “age-friendly communities” project, and (4) to promote the advancement of positive aging.

## 2. Literature Review and Hypotheses

### 2.1. Effects of Neighborhood Environment on Elderly Health

Some scholars considered that the neighborhood environment should include both the physical environment and interpersonal environment [16]. The physical environment reflects the physical infrastructure of a neighborhood, which mainly involves the greening condition, barrier-free roads, fitness facilities, etc. [32], while the interpersonal environment reflects the interaction between neighbors [33].

The relationship between the physical environment and elderly health has been studied extensively [14,34,35]. Chen, While, and Hicks [15] found that neighborhoods with adequate sport facilities were more likely to promote elderly health. Yan & Gao [36] confirmed the significant effect of the physical environment on mental health of the elderly. Wiles et al. [37] pointed out that the physical environment was an important factor affecting the life quality of older adults. Menec and Nowicki [22] and Dunstan et al. [38] confirmed a linear relationship between the physical environment and health of the elderly.

Meanwhile, the significant effects of the interpersonal environment on elderly health have also been verified by some studies [16,26]. Kim and Kawachi [39] found that neighborhoods with a good interpersonal environment can enable older adults to participate more in social interactions, and spread and share more information conducive to their health. Troutman et al. [40] believed that an interpersonal environment can provide more emotional support, which effectively promotes their health. Spring [16] indicated that a good interpersonal environment has a significant long-term impact on elderly health. Lehning, Smith, and Dunkle [35] considered that trust and support among the members of a neighborhood can effectively reduce depression. Cramm and Nieboer [41] even believed that emotional and instrumental support from a good interpersonal environment are important predictors of well-being for older people. In contrast, living in neighborhoods with a poor interpersonal environment will have adverse effects on elderly health [33], and even lead to depressive symptoms [26].

Few of these studies were carried out in China and it is not clear whether these effects differ among the elderly at different ages. Therefore, we put forward the following hypotheses:

**Hypothesis** **1a** **(H1a):** *Physical environment has a positive effect on elderly health.*

**Hypothesis** **1b** **(H1b):** *Interpersonal environment has a positive effect on elderly health.*

### 2.2. Effects of Social Participation on Elderly Health

Plenty of empirical studies have shown that the degree of participation in social activities is closely related to both the mental and the physical health of older adults. On the one hand, social participation is beneficial to the mental health of the elderly. Morrow-Howell et al. [42] suggested that both formal and informal participation in activities can help the elderly to spread their experience and wisdom and gain more respect and recognition. It has also been pointed out that social participation can effectively reduce depressive symptoms of older adults [17,18]. Ichida et al. [43] examined the significant impact of social participation on self-rated health of the elderly. On the other hand, social participation promotes the physical health of the elderly. Morrow-Howell and Gehlert [44] found that the elderly involved in more social activities were stronger than those who do not, possibly due to the more opportunities for them to exercise. Miller and Weissert [45] and Menec et al. [28] showed that social participation can significantly reduce the impairment of physical function of older people. The studies by Kawachi, Kennedy, and Glass [46] and Glass et al. [47] showed that social participation can even reduce the risk of death. In addition, Vogelsang [19] systematically analyzed urban–rural differences in the relationship between social participation and elderly health.

While it is certainly true that social participation can promote elderly health, studies based on Chinese data are few in number. Meanwhile, whether there are differences among older people of different age stages has not been explored. Hence, the following hypothesis was proposed:

**Hypothesis** **2** **(H2):** *Enhanced social participation can effectively promote elderly health.*

### 2.3. Effects of Neighborhood Environment on Social Participation of the Elderly

Environment plays a vital role in the lifestyle of human beings [48]. Structure theory endows environment with social meaning and holds that human behaviors and social interaction always occur in a specific spatial environment. People choose different types of activity in different environments. Both a high-quality physical environment [13,49,50,51] and good interpersonal environment can effectively promote social participation of older adults [10,11,12,13]. Thus, we put forward the following hypotheses:

**Hypothesis** **3a** **(H3a):** *Physical environment has a positive effect on social participation of the elderly.*

**Hypothesis** **3b** **(H3b):** *Interpersonal environment has a positive effect on social participation of the elderly.*

### 2.4. Neighborhood Environment, Social Participation, and Elderly Health

As “age-friendly communities” has been highly recognized by international societies, more and more scholars believe that neighborhoods share the characteristics of ecosystems [21,52]. The relationship between the neighborhood environment and elderly health is not simply linear, but involves the mediating effects of the behavior of older adults. It emphasizes the interaction between neighborhood environment, behavior, and health. For the elderly living in neighborhoods, the process of the environmental impact on their health is a complicated path from neighborhood environment to behavior and then to health. It requires multi-disciplinary and multi-dimensional studies based on a social ecological model.

**Figure 1:**
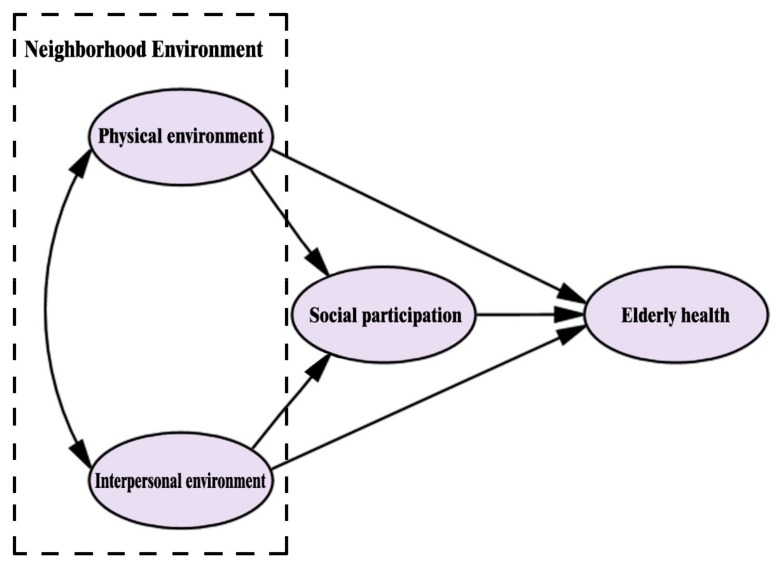
Construction of the concept model.

However, studies that have employed social participation as a mediating variable of environment impact on health are rare. Lehning, Smith, and Dunkle [35] proposed that the relevant studies should link the neighborhood environment with social participation. Therefore, based on the theory of social ecology, we constructed a conceptual model of “neighborhood environment–social participation–elderly health,” as shown in Figure 1. We believe that the effects of neighborhood environment on elderly health is not independent, and the role of social participation cannot be ignored. On this basis, the following hypotheses were put forward:

**Hypothesis** **4a** **(H4a):** *Social participation is a mediator of the relationship between physical environment and elderly health.*

**Hypothesis** **4b** **(H4b):** *Social participation is a mediator of the relationship between interpersonal environment and elderly health.*

## 3. Methods

### 3.1. Data and Sample

Our study was based on a survey conducted in 2014 of the 2839 people aged 60 or above who live in the neighborhoods in Xinhua Subdistrict, Changning District, Shanghai. This survey was to study the relationships between environment, behavior, and elderly health.

Shanghai, located in east China, is a center of economy, finance, trade, shipping, and technological innovation. Xinhua Subdistrict in Changning District is located in the central area of Shanghai, with rich cultural and educational resources, and comfortable living environment. The population of this subdistrict (about 2.2 km^2^) is about 78,000 belonging to 198 neighborhoods of 17 residential districts, 16% of which aged 65 and above.

**Figure 2:**
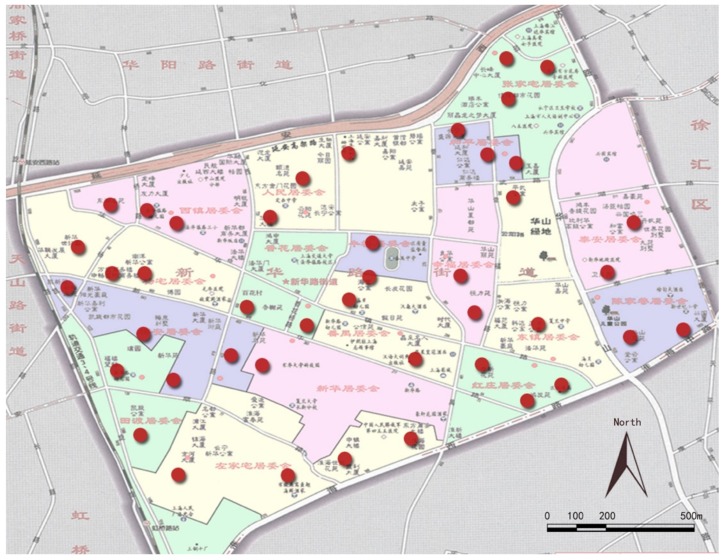
Map of the neighborhood samples.

This survey was carried out in two stages. The first stage was neighborhood sampling. As shown in Figure 2, 43 neighborhoods were selected for sampling according to the geographical locations, year of completion, etc. The second stage was the sampling of the elderly. At this stage, a list of elderly volunteers aged 60 or above from each of the 43 neighborhoods was drawn first. If there were more than 120 samples aged 60 or above in a selected neighborhood, 120 samples without cognitive impairment were randomly selected for the survey. If less than 120, all the older adults without cognitive impairment were selected as samples. After excluding the invalid samples, a total of 2783 valid samples were obtained.

These samples were divided into three groups according to age: low-age group (aged 60–69), middle-age group (aged 70–79), and high-age group (aged 80 or above). As a result, there were 1292 low-age samples, 964 middle-age samples, and 527 high-age samples.

The ethical approval code number is IRB#2015-12-0574 from the Institutional Review Board of the School of Public Health at Fudan University.

**Figure 3:**
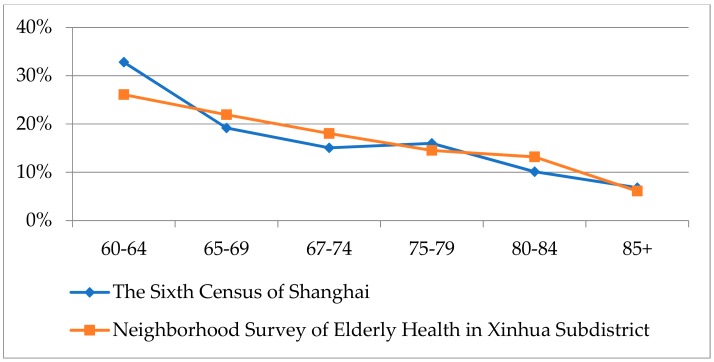
Comparison of age distributions.

We compared the age distribution of people aged 60 and above in Shanghai in the Sixth Census (2010) with that of the samples in this survey. The age structure of the two was basically the same, so we believe that the samples of this survey can represent all the elderly in Shanghai well, as shown in Figure 3.

### 3.2. Measurement

#### 3.2.1. Dependent Variable: Elderly Health

We used a score of self-rated health (SRH) to measure the dependent variable, namely elderly health. SRH has been widely used in the self-evaluation of one’s overall health condition [53,54]. It can effectively measure health conditions, times of receiving medical advice, and mortality [55], and has been considered as a good predictor of objective health conditions that is even more important than actual medical measurements [56,57]. In this study, a subjective health assessment was used to measure the elderly health. All the items of SRH had five options from 1 to 5, with a higher option value indicating better health condition.

#### 3.2.2. Independent Variable: Neighborhood Environment

Scales of aesthetic quality and neighborhood interaction [58] were used to measure physical environment and interpersonal environment, respectively.

Physical environment included six items: “There are many opportunities for us to do exercise in our neighborhood,” “Walking in our neighborhood is very enjoyable,” “There is plenty of tree shade in our neighborhood,” “There are a lot of people walking in our neighborhood,” “There are many people taking exercise in our neighborhood,” and “There are many sports facilities in our neighborhood.” The responses for each item ranged from 1 to 5 (1 = strongly disagree, 2 = disagree, 3 = neutral, 4 = agree, and 5 = strongly agree). The higher score indicates a higher recognition degree of the physical environment.

Interpersonal environment included five items: “People of our neighborhood would like to help each other,” “I often participate in activities with people in our neighborhood,” “I often communicate with people in our neighborhood about personal matters,” “I often help people in our neighborhood to take care of his house or property when he is not at home,” and “I often talk to people in our neighborhood at home or on the street.” The score from 1 to 4 (1 = never, 2 = rarely, 3 = sometimes, and 4 = often) for each item represents the frequency of neighborhood interaction.

#### 3.2.3. Mediating Variable: Social Participation

At present, the definition of social participation of the elderly is not unified. Some scholars believe that social participation of older adults included all the social activities and productive activities in social interaction [59], while others consider it to include personal actions and contributions to others [60]. Our study defined it as various activities in which the older adults participate in their neighborhood, including five styles: volunteer works, self-management and mutual assistance activities, lectures and reports, recreational and sports activities, and interest groups. The degree of social participation was assessed by asking the respondents how often they had participated in various activities in the past 12 months. Responses for each item ranged from 1 to 5 (1 = never, 2 = several times every year, 3 = several times every month, 4 = once a week, and 5 = two to three times every week). A higher score means a higher degree of social participation.

#### 3.2.4. Control Variables

Income and education were employed as the control variables in this study. Responses for each item of monthly income ranged from 1 to 6 (1 = <1500 yuan, 2 = 1500–2500 yuan, 3 = 2500–3500 yuan, 4 = 4500–5500 yuan, 5 = 4500–5500 yuan, and 6 = >5500 yuan), and those of education ranged from 1 to 5 (1 = middle school and below; 2 = high school, special school, or technical school; 3 = junior college; 4 = regular college; and 5 = master’s and above).

### 3.3. Analysis

Descriptive statistical analysis and structural equation modeling (SEM) were used in this study. The SEM method has advantages in the quantitative study of multi-variable interaction and group comparison. Therefore, we used SEM to analyze the complex relationship between neighborhood environment, social participation, and elderly health in different age groups. In order to test whether the data fit the SEM analysis, we grouped all observed variables with 27 and 73 quartiles as critical values and performed a *t*-test. The results showed that all variables had good discrimination and were suitable for SEM analysis. Moreover, the sample size was 2783 (>1000), which can be regarded as approximately following a normal distribution.

Confirmatory factor analysis was carried out on the measurement models of physical environment, interpersonal environment, and social participation. According to the results, for every measurement model, the value of composite reliability (CR) was above 0.6, and the value of average variance extracted (AVE) was above 0.5. The factor loadings of all observation variables were above 0.6 and the reliability coefficients were above 0.36 [61]. It indicated that all measured models had good reliability and validity, and were suitable for SEM analysis.

The model fitting results showed that GFI (goodness-of-fit index) and RMSER (root-mean-square error of approximation) met the ideal criterion, but the X^2^/DF (ratio of chi-square to degree of freedom), AGFI (adjusted goodness-of-fit index), IFI (incremental fit index), and CFI (comparative fit index) did not. Therefore, model optimization was necessary. The fitting output showed that the value of revised index between residual e9 of observed indicator IE1 and residual e10 of observed indicator IE2 was at a maximum. Redefinition of their co-variation could reduce the chi-square value by 60.315 at least. Hence, model fitting was carried out again after e9 was associated with e10. However, X^2^/DF, IFI, and CFI still did not meet the ideal criterion, and further model optimization was required. After establishing three co-variation relations between e5 and e6, e6 and e7, e10 and e11, X^2^/DF, IFI, and CFI finally met the ideal criterion. After optimization, the models were of good fitness, as shown in Table 1.

## 4. Results

### 4.1. Descriptive Statistics of Samples

Table 2 shows descriptive statistics of the main variables. The mean values of the observable variables of physical environment had no significant difference among different age groups. However, the mean values of interpersonal environment and social participation were lower in the high-age group than in the middle-age and low-age groups. The SRH of older adults gradually decreased with the increase of age.

### 4.2. Analysis of Fitting Results of Full Sample Models

**Figure 4:**
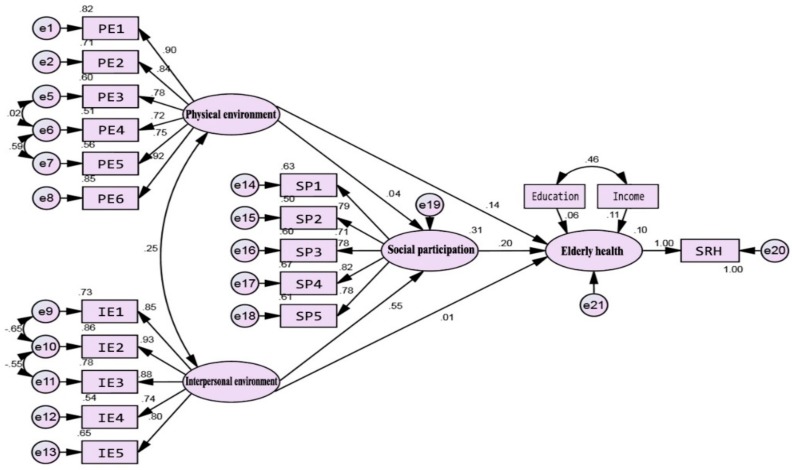
Standardization coefficients for full sample model.

When mediating variables exist in the model, the relationship between independent variables and dependent variables should be expressed as total, direct, and indirect effects [62]. Table 3 and Figure 4 show the results of the full sample model.

All the total effects of the physical environment, interpersonal environment, and social participation on elderly health were significant. The total effect values, from high to low, were social participation, the physical environment, and interpersonal environment in turn. The direct effect of the physical environment on elderly health was significant, but the indirect effect was not, indicating there was no mediating effect. In contrast, the indirect effect of the interpersonal environment on elderly health was significant, but the direct effect was not, indicating a completed mediation effect existed. It suggested that the positive effects of the interpersonal environment on elderly health were completely realized through social participation. In addition, the social participation of the elderly was only affected by the interpersonal environment. Hence, hypotheses 1a, b, 2, 3b, and 4b, but not hypotheses 3a and 4a, were accepted in the full sample model.

### 4.3. Comparison of the Model Paths in Different Age Groups

#### 4.3.1. Significance Tests of Group Differences

First, without considering the values of factor loading, the null hypothesis was proposed as one where the path coefficients of all three group models were equal. The results of the significance test showed that the null hypothesis was rejected, indicating that at least one group difference of path coefficients existed. According to the parameter matrix, the differences of group models were mainly reflected in three paths: social participation to elderly health, physical environment to elderly health, and interpersonal environment to social participation (z-value was above 1.96). In general, the effects of the neighborhood environment and social participation on elderly health were significantly different among the age groups.

#### 4.3.2. Paths Differences in Group Models

By comparing the fitting outputs of different group models, we clearly found differences between various paths and effects among the older adults in different age stages. Table 4 shows the estimated coefficients for the three group models.

According to the total effects on elderly health from different age groups, the effect of the physical environment on elderly health decreased with age, which was significant for the low-age and middle-age groups, but not for the high-age group. In contrast, the effects of interpersonal environment and social participation on elderly health increased with age, which was significant for the high-age and middle-age groups, but not for the low-age group. In terms of the indirect and direct effects on elderly health (only the groups with significant total effect were considered), the physical environment had a significantly direct effect but an insignificant indirect effect, indicating that the mediating effect did not exist. On the contrary (only the groups with significant total effect were considered), the interpersonal environment had a significant indirect effect on elderly health but an insignificant direct effect, indicating a complete mediating effect. That is to say, the positive effect of interpersonal environment on elderly health was completely realized through social participation.

For the elderly in the low-age group, their health was only affected by physical environment. Meanwhile, interpersonal environment but not physical environment had a significant effect on social participation. Hence, in the low-age model, hypotheses 1a and 3b, but not 1b, 2, 3a, and 4a, b, were accepted. As for the middle-age elderly, their health was influenced by both the physical environment and interpersonal environment, as well as social participation. To be specific, the effect of interpersonal environment on social participation was significant, but that of the physical environment was not. Therefore, for the middle-age model, hypotheses 1a, b, 2, 3b, and 4b, but not 3a and 4a, were accepted. In terms of the high-age group, elderly health was only affected by the physical environment and interpersonal environment, both of which had significant effects on social participation. Hence, in the high-age model, hypotheses 1b, 2, 3a, b, and 4b were accepted, but hypotheses 1a and 4a were not, as shown in Table 5.

## 5. Discussion

Based on the data from a survey on the older adults of 43 neighborhoods in Shanghai, this study analyzed the different relationships between health of the elderly, the neighborhood environment, and social participation among the elderly of different age groups. The results based on all the samples of older adults accept most of the hypotheses. First, it confirmed that the physical environment, interpersonal environment, and social participation all have significant effects on elderly health, which was consistent with some previous studies [15,16,19]. Second, the conclusion that interpersonal environment significantly contributes to the social participation of older adults corroborated some previous studies [10,13]. More importantly, our study verified that social participation had a greater impact on elderly health than the physical environment and interpersonal environment. Moreover, the effect of the interpersonal environment on elderly health was completely realized through the mediating role of social participation, suggesting that social participation plays an extremely important role on elderly health. This importance is not only reflected in direct effect of social participation itself on elderly health, but also in the path of the neighborhood environment affecting elderly health. However, our findings did not accept the hypothesis that physical environment has a significant effect on social participation of older adults, which is inconsistent with the conclusions of Richard, Gauvin, Gosselin, and Lalforest [49] and Kahlert [51]. This discrepancy may have resulted from the different samples and the difference in the measuring modes of neighborhood environment.

The relationship between the neighborhood environment, social participation, and elderly health was significantly different among various age groups. In the low-age model, only hypotheses 1a and 3b were accepted and the health of the older adults of this group was only affected by the physical environment. Regarding the middle-age model, hypotheses 1a,b, 2, 3b, and 4b were accepted; therefore, the health of the middle-age older adults was affected by the physical environment, interpersonal environment, and social participation. Hypotheses 1b, 2, 3a,b, and 4b were accepted in the high-age model. The health of the high-age older adults was affected by the interpersonal environment and social participation. The effect of the physical environment on elderly health decreased with an increase of age, while that of the interpersonal environment and social participation increased with age. The significance of this result is not only in discovering the differences and regularities of elderly health at different ages affected by neighborhood environment and social participation, but also to verify the necessity of group studies on the health of the elderly by age. As Germain, Vasquez, Batsis, and Mcquoid [63] pointed out, the studies that ignore the effects of age differences can lead to bias or even errors in conclusions about the well-being of older adults.

To improve the overall health of the elderly, it is necessary to put forward targeted advice and strategies according to the characteristics of different age groups. For the low-age elderly, improving the external physical environment should be the focus in order to promote their health. For the middle-age elderly, not only the improvement of the physical environment and interpersonal environment, but also the increase of the frequency of their social participation is needed. As for the high-age elderly, they have more urgent needs for a better interpersonal environment and more social participation. Therefore, in order to effectively improve the health status of older adults, we should actively guide and organize activities that can promote social participation while improving the interpersonal environment.

We should not only pay attention to health care, pension, insurance, etc., but also focus on the construction of age-friendly communities. Governments should put more emphasis on the building of better recreational and interaction spaces, forming better neighborhood interaction atmospheres, and organizing more social activities.

We recognize some deficiencies in our study. First, as the survey only covered the samples from the Xinhua Subdistrict, Changning District, Shanghai, the neighborhood samples cannot represent all neighborhood environments in China. Second, although the neighborhoods were selected based on the diversity of geographical locations and construction periods, completely systematic random sampling of older adults was not realized, leading to uncertainty of the sample structure.

We will continue the research and improve it from two aspects. First, expanding the survey area and enlarging the sample size to make the findings more representative and applicable. Second, with the reconstructions of some communities in Shanghai for aging, we will carry on a comparative study before and after the reconstructions, which can be more helpful to further discuss the relationship between the neighborhood environment and elderly health.

## 6. Conclusions

It has been a rapidly growing topic in both practical and academic domains to help older adults obtain more social participation opportunities and higher health level through the construction of age-friendly communities. While some valuable studies have demonstrated the positive effects of the neighborhood environment and social participation, they have not sufficiently revealed the complex mechanisms of the interaction among the neighborhood environment, social participation, and elderly health. This study employed social participation as a mediating variable to explore the impact of the neighborhood environment on elderly health. With the increase of age, the positive effect of the physical environment on elderly health was gradually reduced, while that of the interpersonal environment and social participation were gradually strengthened.

In summary, this study not only identified the important role of social participation in the relationship between the neighborhood environment and elderly health, but also explored the differential effects of the physical environment, interpersonal environment, and social participation on the health of the elderly in different age groups. Therefore, it is necessary to provide targeted suggestions and strategies according to the characteristics of different groups. Differential policies and opinions should be applied in improving the interpersonal environment, optimizing physical environment, organizing community activities, etc.

## Figures and Tables

**Table 1:** Comparison of the fitting indexes before and after model optimization.

	GFI	AGFI	IFI	CFI	RMSEA	X^2^/DF
Pre-optimization model	0.910	0.876	0.852	0.849	0.037	5.135
Post-optimization model	0.938	0.936	0.905	0.904	0.028	3.507
Ideal standard	>0.9	>0.9	>0.9	>0.9	<0.08	<5.0

**Table 2:** Description of main variables.

Variable Names	Observed Indicators	Content of the Questions	Mean (All)	Mean (Low-Age)	Mean (Middle-Age)	Mean (High-Age)
Physical environment	PE1	There are many opportunities for us to do exercise in our neighborhood.	3.00	3.02	2.94	3.05
	PE2	Walking in our neighborhood is very enjoyable.	3.31	3.33	3.28	3.32
	PE3	There is plenty of tree shade in our neighborhood.	3.14	3.11	3.11	3.25
	PE4	There are a lot of people walking in our neighborhood.	3.32	3.27	3.32	3.45
	PE5	There are many people taking exercise in our neighborhood.	3.18	3.16	3.17	3.27
	PE6	There are many sports facilities in our neighborhood.	2.95	2.97	2.89	2.98
Interpersonal environment	IE1	People of our neighborhood would like to help each other.	2.37	2.45	2.38	2.13
	IE2	I often participate in activities with people in our neighborhood.	2.15	2.18	2.25	1.87
	IE3	I often communicate with people in our neighborhood about personal matters.	2.34	2.51	2.35	2.04
	IE4	I often help people in our neighborhood to take care of his house or property when he is not at home.	1.91	1.96	1.91	1.77
	IE5	I often talk to people in our neighborhood at home or on the street.	2.44	2.51	2.49	2.17
Social participation	SP1	I often take part in volunteer work of our neighborhood.	1.64	1.72	1.68	1.23
	SP2	I often join in groups of mutual-help or self-management of our neighborhood.	1.50	1.45	1.53	1.38
	SP3	I often listen to report or lecture in our neighborhood.	1.65	1.65	1.71	1.43
	SP4	I often take part in activities of sports or cultural of our neighborhood.	1.76	1.77	1.77	1.41
	SP5	I often take part in outdoor interest groups of our neighborhood.	1.87	1.92	1.89	1.47
Control variables	Income	My family’s monthly per capita income level	3.33	3.30	3.42	3.22
	Education	My level of education	2.24	2.17	2.51	1.95
Elderly health	SRH	My assessment of my health level	2.35	2.52	2.26	2.08

**Table 3:** Total, direct, and indirect effects of the full sample model.

Independent Variable	Mediating Variable (Social Participation)	Dependent Variable (SRH of Older Adults)		
		Total Effect	Direct Effect	Indirect Effect
Physical environment	0.025	0.141 **	0.136 **	0.005
Interpersonal environment	0.547 ***	0.115 **	0.005	0.110 **
Social participation	-	0.201 **	0.201 **	-

Notes: *** means significance at the 0.01 confidence level; ** means significance at the 0.05 confidence level.

**Table 4:** Results of three group models.

Variable Names	Groups	Social Participation (Mediator)	Elderly Health (Dependent Variable)		
			Total Effect	Direct Effect	Indirect Effect
Physical environment	Low-age	−0.040	0.196 **	0.194 **	−0.002
	Middle-age	0.019	0.118 **	0.113 **	0.005
	High-age	0.124 **	0.080	0.033	0.047
Interpersonal environment	Low-age	0.540 ***	−0.052	−0.083	0.031
	Middle-age	0.524 ***	0.124 **	−0.014	0.138 **
	High-age	0.483 ***	0.208 **	0.024	0.183 **
Social participation	Low-age	-	0.058	0.058	-
	Middle-age	-	0.264 **	0.264 **	-
	High-age	-	0.380 ***	0.380 ***	-

Notes: *** means significance at the 0.01 confidence level; ** means significance at the 0.05 confidence level.

**Table 5:** Test results of Hypotheses.

Hypotheses		Results (Accepted or Not)			
		All Samples	Low-Age	Middle-Age	High-Age
1	1a: Physical environment has a positive effect on elderly health.	Yes	Yes	Yes	No
	1b: Interpersonal environment has a positive effect on elderly health.	Yes	No	Yes	Yes
2	Enhanced social participation can effectively promote elderly health.	Yes	No	Yes	Yes
3	3a: Physical environment has a positive effect on social participation of older adults.	No	No	No	Yes
	3b: Interpersonal environment has a positive effect on social participation of older adults.	Yes	Yes	Yes	Yes
4	4a: Social participation is a mediator of the relationship between physical environment and elderly health.	No	No	No	No
	4b: Social participation is a mediator of the relationship between interpersonal environment and elderly health.	Yes	No	Yes	Yes
